# An international survey on AI in radiology in 1041 radiologists and radiology residents part 2: expectations, hurdles to implementation, and education

**DOI:** 10.1007/s00330-021-07782-4

**Published:** 2021-05-11

**Authors:** Merel Huisman, Erik Ranschaert, William Parker, Domenico Mastrodicasa, Martin Koci, Daniel Pinto de Santos, Francesca Coppola, Sergey Morozov, Marc Zins, Cedric Bohyn, Ural Koç, Jie Wu, Satyam Veean, Dominik Fleischmann, Tim Leiner, Martin J. Willemink

**Affiliations:** 1 Department of Radiology, University Medical Center Utrecht, Utrecht, The Netherlands; 2 Department of Radiology, Elisabeth-TweeSteden Ziekenhuis, Tilburg, The Netherlands; 3 Department of Radiology, University of British Columbia, Vancouver, Canada; 4 Department of Radiology, Stanford University School of Medicine, Stanford, CA USA; 5 Department of Radiology, Motol University Hospital, Prague, Czech Republic; 6 Department of Radiology, University Hospital of Cologne, Cologne, Germany; 7 Department of Radiology, IRCCS Azienda Ospedaliero-Universitaria di Bologna, Bologna, Italy; 8 Department of Health Care of Moscow, Research and Practical Clinical Center of Diagnostics and Telemedicine Technologies, Moscow, Russia; 9 Department of Medical Imaging, Saint Joseph Hospital, Paris, France; 10 Department of Radiology, UZ Leuven, Leuven, Belgium; 11 Section of Radiology, Ankara Golbasi Sehit Ahmet Ozsoy State Hospital, Ankara, Turkey; 12 Department of Civil and Environmental Engineering, Stanford University, Stanford, CA USA; 13 Department of Radiology, UT Southwestern Medical Center, Dallas, TX USA

**Keywords:** Radiology, Diagnostic imaging, Artificial intelligence, Surveys and questionnaires

## Abstract

**Objectives:**

Currently, hurdles to implementation of artificial intelligence (AI) in radiology are a much-debated topic but have not been investigated in the community at large. Also, controversy exists if and to what extent AI should be incorporated into radiology residency programs.

**Methods:**

Between April and July 2019, an international survey took place on AI regarding its impact on the profession and training. The survey was accessible for radiologists and residents and distributed through several radiological societies. Relationships of independent variables with opinions, hurdles, and education were assessed using multivariable logistic regression.

**Results:**

The survey was completed by 1041 respondents from 54 countries. A majority (*n* = 855, 82%) expects that AI will cause a change to the radiology field within 10 years. Most frequently, expected roles of AI in clinical practice were second reader (*n* = 829, 78%) and work-flow optimization (*n* = 802, 77%). Ethical and legal issues (*n* = 630, 62%) and lack of knowledge (*n* = 584, 57%) were mentioned most often as hurdles to implementation. Expert respondents added lack of labelled images and generalizability issues. A majority (*n* = 819, 79%) indicated that AI should be incorporated in residency programs, while less support for imaging informatics and AI as a subspecialty was found (*n* = 241, 23%).

**Conclusions:**

Broad community demand exists for incorporation of AI into residency programs. Based on the results of the current study, integration of AI education seems advisable for radiology residents, including issues related to data management, ethics, and legislation.

**Key Points:**

*• There is broad demand from the radiological community to incorporate AI into residency programs, but there is less support to recognize imaging informatics as a radiological subspecialty*.

*• Ethical and legal issues and lack of knowledge are recognized as major bottlenecks for AI implementation by the radiological community, while the shortage in labeled data and IT-infrastructure issues are less often recognized as hurdles*.

*• Integrating AI education in radiology curricula including technical aspects of data management, risk of bias, and ethical and legal issues may aid successful integration of AI into diagnostic radiology*.

## Introduction

In Part 1 of the “International survey on AI in radiology,” we saw that fear of being replaced by artificial intelligence (AI) is still present among radiologists, although an open and more proactive attitude regarding the clinical adoption of AI can also be expected in a substantial proportion of radiology residents and radiologists [1]. AI-specific knowledge turned out to be an important influencing factor, with either no AI-specific knowledge or an intermediate to advanced knowledge level decreasing fear and increasing an open and proactive attitude. Limited AI-specific knowledge resulted in increasing fear and decreasing levels of open and proactive attitude.

Apart from individual factors, organizational factors also play a role when intending to adopt a new technology. A recently emerging topic in the scientific literature is bottlenecks and facilitating factors concerning the implementation of AI applications in diagnostic radiology [2–7]. Commonly identified hurdles by experts are ethical and regulatory issues, work-flow integration, and cost-effectiveness. Hurdles to implementation anticipated by the general radiology community, including non-AI experts, might be similar, but remain currently unknown.

Apart from personal, organizational, and ethical factors, certain technical aspects pertaining to the process of algorithm development could be hurdles as well. Examples include the lack of high-quality images and high-quality labeled data, and the lack of external validation hindering the generalization of algorithms [6, 8]. Currently, it is not clear whether the radiology community at large is aware of these pitfalls.

Furthermore, controversy exists if and to what extent AI should be incorporated into residency programs, while in Part 1 we have shown that intermediate to advanced AI knowledge is associated with an open and proactive attitude towards AI [1]. In this study, we sought to explore the expectations regarding the implementation of AI in radiological practice, including the anticipated hurdles, as well as the opinion of the radiological community concerning the incorporation of AI education in residency programs.

## Materials and methods

### Questionnaire

No institutional review board approval was needed. Analysis was done with anonymized data. A webbased survey using Google Forms (Google LLC) was created consisting of 39 questions on demographics, background, social media use, awareness and existing knowledge, expectations of AI, anticipated hurdles to implementation, AI in residency programs and preferred self learning methods regarding AI. In Appendix 1 of Part 1, all survey questions including their multiple-choice options are listed [1]. A pilot was performed with 10 radiologists and residents to eliminate question ambiguity [9]. The survey was then adjusted and translated by native speakers in nine languages (English, French, German, Spanish, Italian, Dutch, Czech, Russian, and Turkish). The survey and a brief cover letter were accessible between April 18 and July 12, 2019, on www.airadiologysurvey.com. Survey distribution was done through Italian, French, and Dutch radiology societies (SIRM, SFR, and NVvR); the European Society of Medical Imaging Informatics (EuSoMII); as well as social media. A detailed description of the survey distribution is given in Part 1 [1].

### Statistical analysis

Continuous data are presented as means with standard deviations or medians with ranges, as appropriate. Categorical data are presented as proportions. The outcomes on questions “Should AI education become part of residency programs?” and “Should AI become part of residency programs?” were dichotomized in “yes” versus “maybe” or “no” for analysis. Associations of independent variables with the outcomes on expected term of impact as well as hurdles to implementation, and incorporation of AI and imaging informatics in radiology curricula were assessed using multivariable logistic regression. Variables (age, gender, region (European versus non-European), type of hospital (academic, non-academic, private), scientific background, current position (resident versus radiologist), professional social media use, knowledge of informatics/statistics, AI-specific knowledge, and subspecialty) were selected beforehand and included in all logistic regression analyses. In outcomes with low number of events, no logistic regression analysis was performed due to limited statistical power. Age was modeled as a continuous variable; all other variables were modeled as categorical variables. A more detailed description of variable handling can be found in Part 1 [1]. Results of the logistic regression analyses are presented as adjusted odds ratios (ORs) with 95% confidence intervals (CI). Statistical analyses were done in IBM SPSS Statistics for Windows (version 24.0, IBM Corp.). A *p* value < 0.05 was deemed statistically significant.

## Results

### Demographics

A total of 1086 respondents completed the survey. Forty-five respondents were excluded because they were not part of the target population (e.g., student, industry, researcher, entrepreneur) or were double entries, resulting in a final population of 1041 respondents from 54 countries. A summary of the demographics of all respondents is given in Table 1. In Part 1, a detailed description of demographics stratified per source population (i.e., SIRM, NVvR, SFR, other) is given [1].

**Table 1:** Baseline characteristics of all respondents (*n*=1041)

Category		*N* (%)
Gender (male)		670 (65%)^a^
Age (median (range))		38 (24–70)
Region	Africa	14 (1%)
	Asia	73 (7%)
	Australia	8 (1%)
	Europe	867 (83%)
	North America	65 (6%)
	South America	14 (1%)
Type of hospital	Academic	471 (45%)
	Non-academic	367 (35%)
	Private	203 (20%)
Current position	Radiologist	692 (66%)
	Fellow	27 (3%)
	Resident	322 (31%)
Subspecialization	Abdominal	328 (32%)
	Musculoskeletal	214 (23%)
	Neuro	208 (20%)
	Interventional	183 (18%)
	Breast	115 (11%)
	Cardiothoracic	179 (17%)
	Pediatric	89 (9%)
	Molecular/nuclear	41 (4%)
Advanced scientific background^b^	No	727 (70%)
	PhD	148 (14%)
	Research fellowship	51 (5%)
	PhD & research fellowship	23 (2%)
	Obtaining PhD/research fellowship	92 (9%)
Social media use (professional)	No	477 (46%)
	Yes	564 (54%)
	LinkedIn	360 (64%)
	Twitter	115 (20%)
	Instagram	99 (18%)
	Facebook	78 (14%)

^a^Prefer not to say (*n* = 14)

^b^In addition to medical school

### Expectations of AI

#### Term of expected impact

Most respondents thought that AI will help to improve diagnostic radiology (*n* = 926, 89%), some maybe (*n* = 108, 10%), and 1% (*n* = 7) disagreed. Most respondents thought that AI will alter the future of radiologists (*n* = 880, 85%), and minorities were of opinion maybe (*n* = 145, 14%) or not at all (*n* = 16, 2%) (Table 2).

**Table 2:** Expectations and anticipated hurdles to implementation (*n* = 1041)

Question	*N* (%)
Can AI help improve diagnostic radiology?	
Yes	108 (10%)
Maybe	926 (89%)
No	7 (1%)
How can AI help diagnostic radiology? (*n* = 1029)^a^	
Second reader	829 (78%)
Workflow optimization	803 (77%)
Partial replacement	493 (47%)
Full replacement	11 (1%)
Workflow optimization only	99 (10%)
Anticipated hurdles to implementation (*n* = 1024)^a^	
Costs of development	363 (35%)
Cost of software itself	400 (38%)
Lack of	
Trust of stakeholders^b^	376 (36%)
Knowledge of stakeholders	584 (56%)
High-quality image data	159 (15%)
High-quality image labels	287 (28%)
Generalizability of the software	410 (39%)
Ethical/legal issues	630 (62%)
Limitations in digital infrastructure	356 (35%)
Other	14 (1%)

^a^Multiple answers possible

^b^Clinicians, staff, or management

**Fig. 1:**
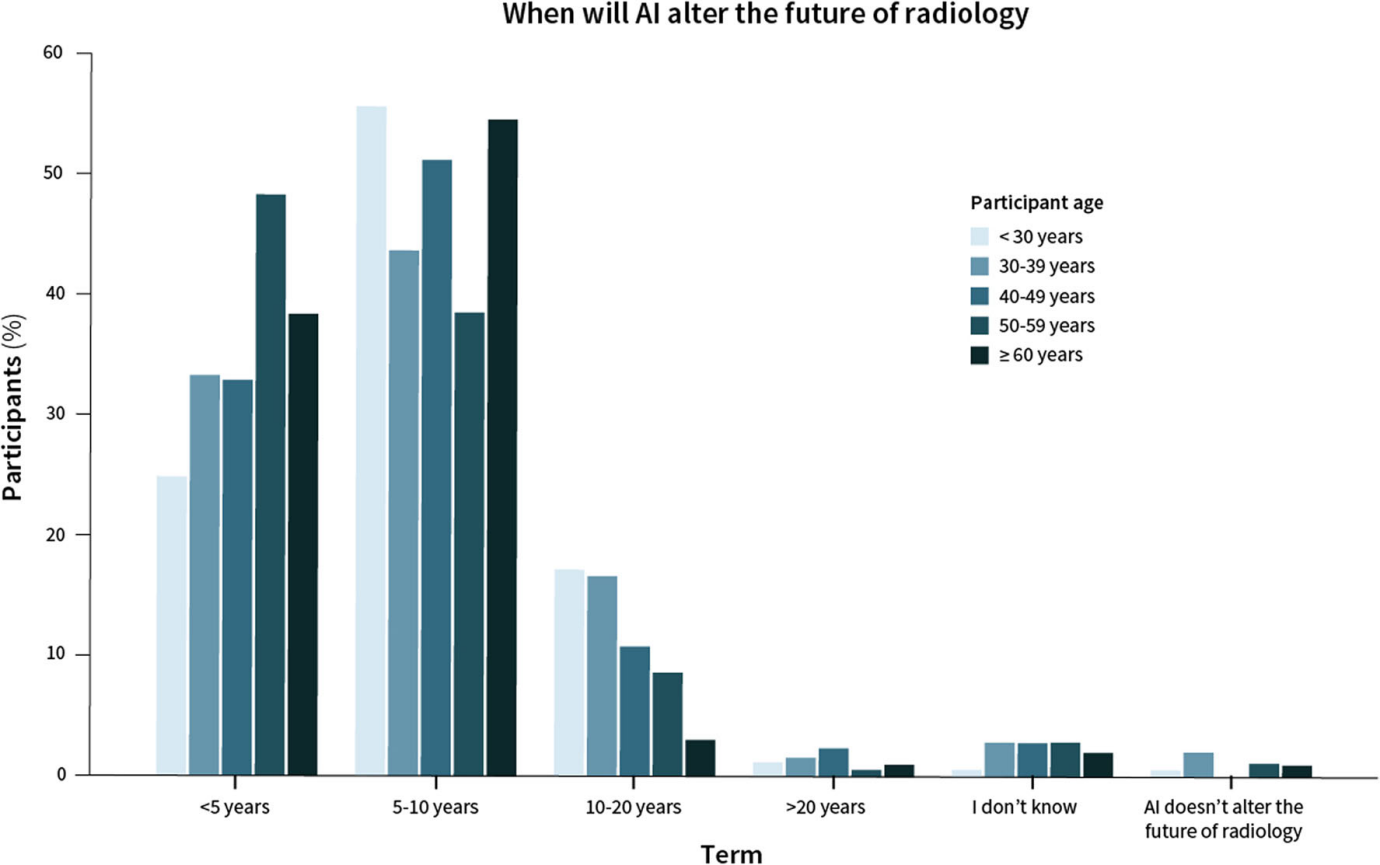
The relation between participant age and when respondents expect AI will alter the radiological clinical setting

The expected term of noticeable effects of AI in radiology was mostly short-term (< 5 years, *n* = 363, 35%) or middle long-term (5–10 years, *n* = 492, 49%). A markable change in > 10 years was expected by *n* = 149 (14%) respondents (Fig. 1).

Independent predictors for expecting change on a short term (< 5 years) were higher age (adjusted OR per 10-year interval 1.26, 95% CI 1.07–1.47, *p* = 0.005), female gender (adjusted OR 1.37, 95% CI 1.02–1.84, *p* = 0.04), respondents who only heard of AI (adjusted OR 2.74, 95% CI 1.14–6.57, *p* = 0.02), and respondents with intermediate (adjusted OR 4.30, 95% CI 1.79–10.26, *p* = 0.001) or advanced AI-specific knowledge (adjusted OR 5.31, 95% CI 2.13–13.23, *p* < 0.001) (Table 3). Respondents with an abdominal subspecialty were less likely to expect change on a short term (adjusted OR 0.69, 95% CI 0.51–0.93, *p* = 0.02).

**Table 3:** Independent predictors for term of expected impact of AI in diagnostic radiology and anticipated hurdles to its implementation

	Independent predictors^a^	Adjusted OR (95% CI)	*p* value
Term of expected impact			
Short term (< 5 years)	Age (10-year interval) Female Heard of AI Intermediate AI-specific knowledge Advanced AI-specific knowledge Abdominal radiologists	1.26 (1.07–1.47) 1.37 (1.02–1.84) 2.74 (1.14–6.57) 4.30 (1.79–10.26) 5.31 (2.13–13.23) 0.69 (0.51–0.93)	*p* = 0.005 *p* = 0.04 *p* = 0.02 *p* = 0.001 *p* < 0.001 *p* = 0.02
Middle long-term (5-10 years)	Male Europe	1.51 (1.14–2.00) 1.69 (1.18–2.42)	*p* = 0.004 *p* = 0.004
Long term (> 10 years)	Age (10-year interval) Europe Social media use Intermediate AI-specific knowledge Advanced AI-specific knowledge	0.64 (0.51–0.82) 0.54 (0.35–0.85) 0.60 (0.41–0.87) 0.23 (0.11–0.52) 0.17 (0.07–0.44)	*p* < 0.001 *p* = 0.008 *p* = 0.008 *p* < 0.001 *p* < 0.001
Anticipated hurdles			
Costs (software or development)	-	-	NS
Lack trust in AI of stakeholders	Europe Cardiothoracic radiologists	0.56 (0.40–0.81) 1.57 (1.11–2.22)	*p* = 0.002 *p* = 0.01
Lack of knowledge or expertise of stakeholders	Private centers	0.63 (0.24–0.94)	*p* = 0.02
Lack of high-quality image data	Europe Private centers Advanced AI-specific knowledge Breast radiologists Pediatric radiologists	0.39 (CI 0.26–0.61) 0.49 (0.27–0.89) 3.37 (1.05–10.84) 0.43 (0.20–0.90) 2.13 (1.20–3.80)	*p* < 0.001 *p* = 0.02 *p* = 0.04 *p* = 0.03 *p* = 0.01
Lack of high-quality image labels	Advanced AI-specific knowledge	5.42 (2.22–13.21)	*p* < 0.001
Lack of generalizability (i.e., external validity)	Age (10-year interval) Europe	0.85 (0.73–0.99) 0.54 (0.38–0.77)	*p* = 0.04 *p* < 0.001
Ethical and legal issues	Europe Basic AI-specific knowledge Intermediate AI-specific knowledge Advanced AI-specific knowledge Musculoskeletal radiologists	0.59 (0.40–0.85) 0.68 (0.48–0.96) 2.90 (1.48–5.65) 2.85 (1.39–5.86) 1.44 (1.03–2.01)	*p* = 0.005 *p* = 0.03 *p* = 0.002 *p* = 0.004 *p* = 0.03
Limitations in digital infrastructure of the hospital/center	Non-academic centers Private centers Abdominal radiologists Cardiothoracic radiologists Interventional radiologists	0.58 (0.42–0.82) 0.57 (0.37–0.87) 1.45 (1.08–1.95) 1.51 (1.05–2.15) 1.55 (1.09–2.21)	*p* = 0.002 *p* = 0.009 *p* = 0.01 *p* = 0.03 *p* = 0.01

^a^Corrected for age, gender, region (European versus non-European), type of hospital (academic, non-academic, private), scientific background, current position (resident versus radiologist), professional social media use, knowledge of informatics/statistics, AI-specific knowledge, and subspecialty

Independent predictors for expecting change on a middle long-term (5–10 years) were male gender (adjusted OR 1.51, 95% CI 1.14–2.00, *p* = 0.004) and working in Europe (adjusted OR 1.69, 95% CI 1.18–2.42, *p* = 0.004) rather than outside of Europe.

Negative predictors for expecting change on the long term (> 10 years) were lower age (adjusted OR 0.64 per 10-year interval, 95% CI 0.51–0.82, *p* < 0.001), working in Europe (adjusted OR 0.54, 95% CI 0.35–0.85, *p* = 0.008), professional social media use (adjusted OR 0.60, 95% CI 0.41–0.87, *p* = 0.008), and intermediate (adjusted OR 0.23, 95% CI 0.11–0.52, *p* < 0.001) or advanced AI-specific knowledge (adjusted OR 0.17, 95% CI 0.07–0.44, *p* < 0.001). There were no differences for hospital type (i.e., academic, non-academic, and private).

#### Expected role of AI in diagnostic radiology on the longer term

The question on the expected role(s) of AI in the longer term was filled out by *n* = 1029/1041 (99%) respondents. Most frequently, the role of AI in the longer term was reported as AI becoming the second reader (*n* = 829/1029, 78%); respondents with advanced AI-specific knowledge were significantly more likely to indicate this (adjusted OR 3.31, 95% CI 1.42–7.69, *p* = 0.006) (Tables 2 and 3).

Partial replacement of radiologists by AI was expected by 47% (*n* = 493/1029) of respondents, and independent predictors were male gender (adjusted OR 1.73, 95% CI 1.30–2.32, *p* < 0.001), intermediate AI-specific knowledge (adjusted OR 2.41, 95% CI 1.12–5.20, *p* = 0.03), and advanced AI-specific knowledge (adjusted OR 4.04, 95% CI 1.78–9.16, *p* = 0.004). Negative predictors for expecting partial replacement were age (adjusted OR per 10-year interval 0.85, 95% CI 0.73–0.99, *p* = 0.04) and abdominal (adjusted OR 0.65, 95% CI 0.49–0.78, *p* = 0.004), breast (adjusted OR 0.48, 95% CI 0.30–0.75, *p* = 0.002), and pediatric (adjusted OR 0.59, 95% CI 0.36–0.98, *p* = 0.04) subspecialties. Full replacement of radiologists by AI was only expected by *n* = 31/1029 (3%) respondents.

Workflow optimization by AI was expected by 77% (*n* = 803/1029) of respondents, and independent predictors were lower age (adjusted OR per 10-year interval 0.77, 95% CI 0.64–0.92, *p* = 0.004) and intermediate (adjusted OR 3.41, 95% CI 1.64–7.09, *p* = 0.001) and advanced (adjusted OR 6.46, 95% CI 2.73–15.31, *p* < 0.001) AI-specific knowledge. Ninety-nine respondents (*n* = 99/1029, 10%) expected that AI will have no image-based role such as detection of pathology (i.e., these respondents expect only workflow optimization).

### Anticipated hurdles to implementation

The question on anticipated hurdles to implementation was filled out by *n* = 1024/1041 (98%) respondents, and respondents could select multiple answers. Indicated hurdles to clinical implementation of AI were mainly ethical and legal issues (*n* = 630, 62%), limitations in digital infrastructure (*n* = 356, 35%), and lack of knowledge (*n* = 584, 56%) of stakeholders (i.e., clinicians, radiology staff, or management) (Table 2).

High costs of AI software development were indicated by *n* = 363/1024 (35%), and high costs of AI software itself were indicated by *n* = 400/1024 (38%); there were no independent predictors for these outcomes (Table 3).

Lack of trust in AI by stakeholders (i.e., clinicians, staff, or management) was reported by *n* = 376/1024 (37%) of respondents, and independently and significantly more often observed in those working outside of Europe (adjusted OR 1.77, 95% CI 1.24–2.53, *p* = 0.002) and cardiothoracic radiologists (adjusted OR 1.57, 95% CI 1.11–2.22, *p* = 0.01).

Lack of knowledge or expertise of stakeholders was reported by *n* = 584/1024 (57%) and significantly less often observed in respondents working in private centers (adjusted OR 0.63, 95% CI 0.24–0.94, *p* = 0.02).

Lack of high-quality image data was reported by *n* = 159/1024 (16%) and significantly less often indicated in respondents working in Europe (adjusted OR 0.39, 95% CI 0.26–0.61, *p* < 0.001), private centers (adjusted OR 0.49, 95% CI 0.27–0.89, *p* = 0.02), and breast radiologists (adjusted OR 0.43, 95% CI 0.20–0.90, *p* = 0.03). This hurdle was more often indicated in respondents with advanced AI-specific knowledge (adjusted OR 3.37, 95% CI 1.05–10.84, *p* = 0.04) and pediatric radiologists (adjusted OR 2.13, 95% CI 1.20–3.80, *p* = 0.01).

Lack of high-quality image labels was reported in *n* = 287/1024 (27%), and significantly more mentioned in those with advanced AI-specific knowledge (adjusted OR 5.42, 95% CI 2.22–13.21, *p* < 0.001).

Lack of generalizability (i.e., external validity) of the software was reported in *n* = 410/1024 (40%), and significantly less mentioned in older respondents (adjusted OR per 10-year interval 0.85, 95% CI 0.73–0.99, *p* = 0.04) and respondents working in Europe (adjusted OR 0.54, 95% CI 0.38–0.77, *p* < 0.001).

Ethical and legal issues were mentioned by *n* = 630/1024 (62%), and significantly more often observed in those working outside of Europe (adjusted OR 1.71, 95% CI 1.17–2.48, *p* = 0.005), those with intermediate (adjusted OR 2.90, 95% CI 1.48–5.65, *p* = 0.002) or advanced (adjusted OR 2.85, 95% CI 1.39–5.86, *p* = 0.004) AI-specific knowledge, and musculoskeletal radiologists (adjusted OR 1.44, 95% CI 1.03–2.01, *p* = 0.03). This hurdle was less often indicated in respondents with basic AI-specific knowledge (adjusted OR 0.68, 95% CI 0.48–0.96, *p* = 0.03).

**Fig. 2:**
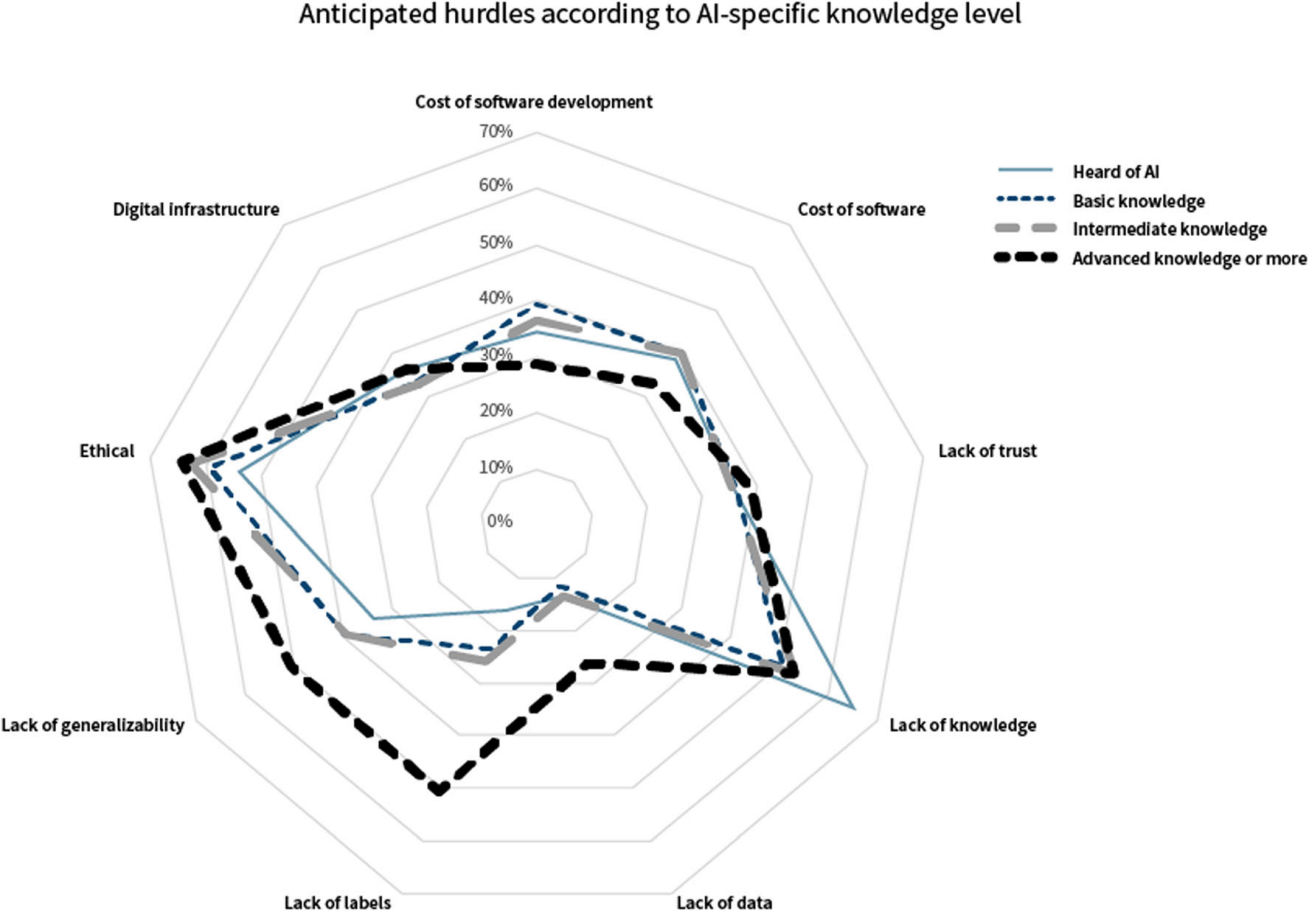
Anticipated hurdles as indicated by respondents according to AI-specific knowledge level

Limitations in digital infrastructure of the hospital/center were mentioned in *n* = 356/1024 (35%) and more often observed in abdominal radiologists (adjusted OR 1.45, 95% CI 1.08–1.95, *p* = 0.01), cardiothoracic radiologists (adjusted OR 1.51, 95% CI 1.05–2.15, *p* = 0.03), and interventional radiologists (adjusted OR 1.55, 95% CI 1.09–2.21, *p* = 0.01). This hurdle was less often indicated in respondents working in non-academic (adjusted OR 0.58, 95% CI 0.42–0.82, *p* = 0.002) or private (adjusted OR 0.57, 95% CI 0.37–0.87, *p* = 0.009) centers, compared to those working in academic centers. Resistance to change was mentioned in open answers by *n* = 7 respondents and lack of radiology-specific knowledge of computer scientists by *n* = 5 respondents. Anticipated hurdles to implementation by AI-specific knowlegde levels are depicted in Fig. 2.

### AI in residency programs

A majority (*n* = 819, 79%) of the respondents indicated that AI education should be incorporated in residency programs, and the remainder indicated maybe (*n* = 182, 18%) or disagreed (*n* = 40, *n* = 4%). Positive predictors for favoring integration of AI education in residency programs were increasing age (adjusted OR 1.43 per 10-year interval, 95% CI 1.20–1.74, *p* < 0.001), being a resident (adjusted OR 1.71, 95% CI 1.09–2.68, *p* = 0.02), only having heard of AI (adjusted OR 2.96, 95% CI 1.48–5.89, *p* = 0.002), intermediate AI-specific knowledge (adjusted OR 3.84, 95% CI 1.90–7.77, *p* < 0.001), and advanced AI-specific knowledge (adjusted OR 5.16, 95% CI 2.33–11.43, *p* < 0.001). Respondents subspecialized in pediatric radiology reported significantly less often they wanted AI education incorporated in residency curricula (adjusted OR 0.58, 95% CI 0.35–0.98, *p* = 0.04) (Table 4).

**Table 4:** Opinions and independent predictors for AI and imaging informatics in radiology curricula (*n* = 1041)

Answers (*n* = %)	Independent predictors^a^	Adjusted OR (95% CI)	*p* value
AI should be incorporated in residency programs			
Yes, *n* = 819 (79%) Maybe, *n* = 182 (18%) No, *n* = 40 (4%)	Age (10-year interval) Residents Heard of AI Intermediate AI-specific knowledge Advanced AI-specific knowledge Pediatric radiologists	1.43 (1.20–1.74) 1.71 (1.09–2.68) 2.96 (1.48–5.89) 3.84 (1.90–7.77) 5.16 (2.33–11.43) 0.58 (0.35–0.98)	*p* < 0.001 *p* = 0.02 *p* = 0.002 *p* = 0.002 *p* < 0.001 *p* = 0.04
AI/imaging informatics should be a subspecialty			
Yes, *n* = 241 (23%) Maybe, *n* = 359 (35%) No, *n* = 437 (42%)	Social media use N/A N/A	1.38 (1.01–1.89) N/A N/A	*p* = 0.04 N/A N/A

^a^Corrected for age, gender, region (European versus non-European), type of hospital (academic, non-academic, private), scientific background, current position (resident versus radiologist), professional social media use, knowledge of informatics/statistics, AI-specific knowledge, and subspecialty

A minority indicated that imaging informatics and AI should (*n* = 241, 23%) or maybe should (*n* = 359, 35%) become a radiology subspecialty, while some (*n* = 437, 42%) disagreed. The only positive predictor for favoring imaging informatics and AI as a subspecialty was professional social media use (adjusted OR 1.38, 95% CI 1.01–1.89, *p* = 0.04).

### Preferred self-learning methods regarding AI

Of respondents, *n* = 780 (75%) responded yes to the question “Are you planning on learning about this topic, even if it’s not a program or CME requirement?”, whereas *n* = 198 (19%) respondents answered “maybe” to this question. *N* = 63 (6%) respondents were not planning to learn about AI [1]. Preferred self-learning media were conferences/specialty courses (*n* = 765, 74%), scientific literature (*n* = 619, 60%), online articles (e.g., on medium or ai.myesr.org) in *n* = 498 (48%), e-learning platforms such as Coursera/EdX (*n* = 448, 43%), and social media including Twitter, LinkedIn, Facebook, and YouTube (*n* = 254, 24%). In general, we found that those participants with intermediate to advanced level AI-specific knowledge are more motivated to use any medium for self-study, and in particular scientific literature and conferences/specialty courses. Table 5 summarizes independent predictors for each medium.

**Table 5:** Independent predictors for self-learning methods pertaining to artificial intelligence in radiology

Self-learning method	Independent predictors^a^	Adjusted OR (95% CI)	*p* value
Scientific literature	Age (10-year interval) Male Social media use Knowledge of statistics/informatics Intermediate AI-specific knowledge Advanced AI-specific knowledge	0.85 (0.72–1.0) 1.56 (1.16–2.09) 1.54 (1.17–2.02) 1.52 (1.15–2.04) 2.54 (1.27–5.07) 5.25 (2.41–11.44)	*p* = 0.04 *p* = 0.003 *p* = 0.003 *p* = 0.004 *p* = 0.008 *p* < 0.001
Conferences or specialty courses	Age (10-year interval) Male Social media use Basic AI-specific knowledge Intermediate AI-specific knowledge Advanced AI-specific knowledge	0.83 (0.70–0.98) 0.71 (0.51–0.98) 1.47 (1.09–1.98) 0.65 (0.44–0.96) 2.86 (1.42–5.75) 3.87 (1.75–8.57)	*p* = 0.03 *p* = 0.04 *p* = 0.01 *p* = 0.03 *p* = 0.003 *p* = 0.001
Online articles (non-scientific)	Social media use Scientific background Advanced AI-specific knowledge	1.63 (1.26–2.13) 0.66 (0.49–0.90) 2.86 (1.38–5.91)	*p* < 0.001 *p* = 0.008 *p* = 0.005
E-learning platforms	Social media use Knowledge of statistics/informatics Basic AI-specific knowledge Advanced AI-specific knowledge Breast radiologists	1.71 (1.31–2.24) 1.37 (1.04–1.82) 0.66 (0.47–0.92) 2.42 (1.14–5.12) 0.49 (0.31–0.77)	*p* < 0.001 *p* = 0.03 *p* = 0.02 *p* = 0.02 *p* = 0.002
Social media	Age (10-year interval) Europe Scientific background Social media use Abdominal radiologist	0.65 (0.52–0.80) 0.43 (0.29–0.67) 0.60 (0.41–0.87) 8.50 (5.60–12.9) 0.63 (0.43–0.91)	*p* < 0.001 *p* < 0.001 *p* = 0.008 *p* < 0.001 *p* = 0.02

^a^Corrected for age, gender, region (European versus non-European), type of hospital (academic, non-academic, private), scientific background, current position (resident versus radiologist), professional social media use, knowledge of informatics/statistics, AI-specific knowledge, and subspecialty

## Discussion

This large (*n* = 1041) international survey of radiologists and residents showed that a majority (*n* = 855, 82%) of participants expects that AI will cause a significant change to the radiology profession within 10 years. Regarding the role of AI in the longer term, a co-pilot setting with AI as second reader and work-flow optimization tasks were most often mentioned, in 78% (*n* = 829) and 77% (*n* = 802) of respondents respectively. When asked about possible hurdles to implementation, ethical and legal issues (*n* = 630, 62%) and lack of knowledge (*n* = 584, 57%) were mentioned most often by all respondents. Respondents with advanced AI-specific knowledge added lack of labelled images and generalizability issues. The majority of respondents (*n* = 819, 79%) wants AI education incorporated in residency programs, while only a minority (*n* = 241, 23%) agrees AI and imaging informatics should be recognized as a subspecialty.

To the best of our knowledge, no large-scale survey has been performed regarding the demand for AI education as part of the radiology residency [10, 11]. Lack of knowledge among stakeholders (i.e., clinicians, staff, or management) was one of the most frequently mentioned hurdles by the respondents. An important step in successful implementation and continuous benefit of AI might therefore be the incorporation of AI education into radiology curricula. In our opinion, this study shows that this is supported by a broad international community.

Similar hurdles to ours were identified by Strohm et al [3], namely the lack of knowledge, finances, and trust among stakeholders, and the ethical and legal aspects.

Respondents with higher AI-specific knowledge levels more commonly identified the lack of labelled and well-organized datasets as a potential issue. This is in line with the general opinion in the current literature [2, 6, 7, 12, 13]. The lack of high-quality labels being a potential hurdle was only indicated by 28% (*n* = 287) of all respondents, which may reflect a relative unawareness of this issue.

Ethical and legal issues were mentioned as a hurdle to implementation in 62% (*n* = 630/1024) of respondents, in line with the expectation of the authors. Therefore, it is possible that a fairly large proportion of respondents does not regard privacy issues and unintentional harm inflicted by algorithms used as a medical device as an issue. This indicates that regulatory issues as well as the different forms of bias and/or potential complications resulting from using algorithms for diagnostic decision-making are topics to be further explored [4] and incorporated into education.

Limitations in digital infrastructure was surprisingly mentioned in a low percentage of respondents (i.e., 35% (*n* = 356)), as seamless work-flow integration is essential for success and it is notoriously difficult to incorporate several software solutions all in a well-integrated IT environment [2].

The majority of respondents (*n* = 780, 75%) indicated to be planning on learning more about AI, while 6% (*n* = 63) was not planning to learn more about AI at all. In this study, we found that younger respondents and those already having intermediate to advanced level AI-specific knowledge are more motivated to use any medium for self-study, and in particular scientific literature and conferences/specialty courses. E-learning platforms and online “grey literature” were also surprisingly popular in this group.

Limitations of the current study were discussed in detail in Part 1 [1] and include mainly a low response rate (3.9%) and selection bias characteristic of survey research. Another limitation, mainly in the identification of anticipated hurdles to implementation, is that these were identified by a single multiple-choice question. Although there was an option for comments, this did not result in valuable additions. Therefore, respondents might have been influenced by these preselected answers. We were not able to verify whether terms as “generalizability” and “digital infrastructure” were understood by the respondents as the researchers intended. Furthermore, some options as identified by Strohm et al [3], like added value in clinical practice, were not included in the options. Regarding the preferred self-learning mediums, this survey was conducted in the pre-COVID-19 era, and probably the online mediums have therefore rapidly gained popularity, and presumably today’s outcomes regarding this subtopic would be very different.

In conclusion, Part 2 of this large international survey shows that there is broad community support for incorporation of AI into residency programs. The radiological community is becoming aware of the hurdles to AI implementation and indicates ethical and legal issues and lack of knowledge as main bottlenecks. It appears that the necessity of well-curated datasets and digital infrastructure are more often overlooked challenges. Based on the results of the current study, integrating AI education in radiology curricula including technical aspects of data management, risk of bias, and ethical and legal issues seems to be the way forward to aid successful integration of AI into diagnostic radiology.

## Declarations

### Guarantor

The scientific guarantor of this publication is Merel Huisman.

### Statistics and biometry

One of the authors has significant statistical expertise.

### Methodology

• cross-sectional study

• multicenter study
